# Intestinal Irrigation—Enteroclysis1Read before the Section on Medicine of the Buffalo Academy of Medicine, February 14, 1893.

**Published:** 1893-04

**Authors:** Frederic W. Bartlett

**Affiliations:** Buffalo, N. Y.


### INTESTINAL IRRIGATION—ENTEROCLYSIS.[1]

BY FREDERIC W. BARTLETT, M. D., Buffalo, N. Y.

IN PRESENTING to the Academy, this evening, the subject of intestinal irrigation, it is not my intention to attempt any description of the anatomy of the large or small intestine, of the pathological changes in disease, or of the various bacilli, more or less prominent, according to modern discoveries, in the causation of acute or chronic organic lesions. But, while I thus dispense with much essential to monographs on important medical subjects, I request my hearers to recall the graphic descriptions of authors, or on reading this essay to turn to Quain, Reynolds, v. Ziemssen, Keating, or other classic works on intestinal diseases, and realize the vast importance of correct treatment. While, primarily, this paper discusses a treatment of dysentery by local medication and manipulation, which I may without offense claim to be largely original with myself, antedating as it does any report with which I am acquainted, of the treatment of dysentery by the special germicidal solution employed, it is intended to apply to all forms of intestinal disease accompanied by mucous, sanguinolent, serous, purulent or septic exudations or secretions from the intestine.

This paper is a summary of reports dating back to the winter of 1883 and 1884 made to the Buffalo Medical and Surgical Association, and at various times since.

In September, 1883, the first case treated occurred, and as it fitly describes the *modus operandi* and results, it will be the only case quoted.

September 8, 1883, I was called to attend George Bauer, then and now residing at 160 Walnut street ; occupation, stone cutter; age, 40 years ; sick for several days with dysentery. I prescribed treatment usual in such cases for several days, but patient grew worse and on the 15th of September had reached a hopeless stage. The evacuations were small, muco-sanguinolent, frequent, painful, exhausting, and patient had received the sacraments in expectation of inevitable death. Although I had ample experience in the treatment of the malady, and had used various enemata in my previous cases with only partial success, it occurred to me upon the theory of germ causation to test in this case the utility of hydrarg. bichloride in solution. I dissolved four grains in four pints of water, and attaching a catheterto a bulb-syringe, passed the entire quantity within the colon. The patient was promptly placed

1. Read before the Section on Medicine of the Buffalo Academy of Medicine, February 14, 1893.

upon a large jar, and the injected fluid and apparently much hitherto retained fecal matter was expelled. Half an hour later another large motion occurred, and no other passage for twenty-four hours, and this painless and greatly improved. Under anodynes and careful diet the patient, in a few days, was well and returned to his work.

My first report to the Association covered sixty-nine cases, a second report 100 more. I have no record of subsequent cases, but think an estimate of a total of 500 enemata not excessive. I have not, for the last few years, waited for the severer symptoms to be manifested. As soon as a diagnosis of colitis or entero-colitis could be made, the enema has been used. When so employed, a single enema has often sufficed. The age of patients treated has ranged from three months to over seventy years. There have been no cases of septicemia or mercurial poisoning; nothing to discourage prompt and thorough use of bichloride in this way.

I consider the mode of operating important and novel as regards details, and thus describe it :

1. Position of patient.

The patient should be placed upon the *right* side, the back, when in this position, turned to the outer edge of the bed or lounge, the hips brought well to the edge of the mattress and the latter protected by an oil-cloth, folded sheet, or quite as well by a few newspapers.

2. The solution may be readily prepared by using one-half of a seven and one-half grain bichloride of mercury tablet (Wyeth's) or an equivalent in other pills or tablets, dissolved in two quarts of warm water. Bichloride in powders suffers oxidation. The receptacle should be a glazed vessel, preferably a pitcher or two-quart glass fruit-jar. Bichloride will attack a metallic surface.

3. The syringe used should be the elastic bulb, with collapsible tube and continuous current and rubber inlets and exits. Of these I prefer the Alpha, on account of the great convenience of joining the catheter to the exit tube. It is only necessary to cut off the bone tip from an eleven or twelve American to permit the easy and perfect connection by slipping the cut end of the tube well within the exit tube of the syringe.

4. Placing the enema reservoir upon a chair in close proximity to the hips of the patient, with the left hand introduce the catheter. The right hand will thus conveniently grasp the bulb of the syringe, and what, without this attention to details, might be a clumsy and awkward performance, will be accomplished without shock to modesty or delay in execution. Introduce the catheter gently onethird or more of its length, and ultimately all of it. It is quite unimportant to pass the sigmoid. I have rarely done so. With patient on the right side, the descending colon becomes horizontal and the transverse, a descending tube, thus aiding by gravitation the passage of the enema to the ileum. Introduce the enema as rapidly as can conveniently be done with due regard to the comfort of the patient, and encourage him to bear patiently the discomfort till the whole amount is injected. As soon as this is done, place the patient upon a large jar or vessel of sufficient capacity to receive the evacuation which may be augmented by retained matters in the colon. If the enema is slow to pass, turn the patient upon the left side and avail of gravity and siphonage of the tranverse colon. If not promptly expelled, restore to right lateral position and use more water, omitting the bichloride. Use an appropriate anodyne by mouth or hypodermatically, rather than a suppository, and treat otherwise *secundem artem.*

I have been thus particular in defining my plan for flushing the intestines, because, recently, many who report cases treated by tannin, boric acid, and other more or less useful agents, simply say they use *enemata.* In most cases the patient is placed on the *left* side, and in books on nursing this position has always been advised. It should be abandoned. Again, as regards the quantity used, it varies from a few ounces to a gallon. By the former allowance only the rectum can be flushed, and Dr. W. W. Johnson, of Washington, D.C., in a paper read in May, 1892, before the American Association of Physicians, in session in Washington, favored this plan. He uses double tubes and thinks it quite difficult to pass tubes beyond the sigmoid. I think the difficulty exaggerated, but as a matter of fact the passage of tubes of any kind is unnecessary. He also objects to the bichloride; boric acid or carbolic acid being preferred. He advises the continuous irrigation of the rectum at intervals at first of three hours, and the treatment is continued some days, the flushing being used at longer intervals. His results are apparently good, but I think the plan I have used dispenses with many troublesome details and is more promptly successful. There can be no advantage in using the rectum as a *cess-pool,* to be emptied at regular intervals, when the whole colon can be emptied at one flushing and the mucous membrane be made thoroughly aseptic. The advantage of instantly terminating a morbid process in the entire colon cannot be overrated. I have objected to enemata of three or four quarts of fluid because of the unnecessary and perhaps dangerous distention of the colon and ileum. It has been shown by Dr. Judson Daland, of Philadelphia, that fluids may be passed post-mortem not only from the rectum to the colon, but also through the ileum, stomach, nose and mouth. While wonderful results may follow large enemata in enteric fever, the inability to diagnose lesions of continuity in the ileum must ever cause the physician to dread induced perforation, possibly from its always fatal results, incurring the penalty of malpractice. As I have previously stated, I have extended the use of germicide enemata to conditions other than dysentery, notably to typhoid fever, of which disease I have treated twenty cases, using enemata practically as *the treatment*, and other medication as adjuvant.

I will here report the first case so treated, and as the result of the other nineteen were successful and practically had the usual distinctive features, it is needless to make detailed reports:

On the 20th of November, 1890, I was called to attend Albert W. Shaw, residing at No. 260 Fulton street. Patient aged about 35 years, was a railroad engineer who had been ill for ten days, but had been advised to keep up exercise. Disease had been diagnosed as nervous dyspepsia. When seen had vital temperature, 105.5°F., pulse 130, so delirious as to require manual restraint, and all the usual symptoms of typhoid fever with rose eruption, abdomen moderately tympanitic, and was said to have had evacuations every fifteen minutes during the day. I deemed it a good case for trying intestinal antisepsis, and flushed the colon with two quarts of warm bichloride solution, 1–8000. The enema and contents of colon were promptly ejected, and in half an hour he had another quite free motion. Patient was given an hypnotic. Morning call, November 21st, had rested better, much less delirium, no evacuation. November 22d, had very good night, no evacuation. Administered enema, 1–8000 bichloride water, two quarts—large, free motion of good quality, delirium gone, temperature, 99°F.; pulse, 80. November 23d, had had a motion, apparently perfectly healthy, good appetite, cheerful, rational, greatly improved. Treatment from this date mainly dietetic, convalescence prompt, no sequelæ.

One year later, I treated Mrs. S. for same disease, convalescing on the fourteenth day. The average duration of the remaining eighteen cases was fourteen days to commencing convalescence. *Diarrhea was always promptly controlled by bichloride enema.* Have treated cases in consultation by same means, with same results. It may be urged that we do not reach the glands of Peyer and Brunner by this plan. This may be conceded, but we do reach the entire surface of the colon, rendering it aseptic and removing all morbid accumulations. This prevents septic absorption and the morbid action of bacilli. The results are the most convincing evidence of the good accomplished.

I have extended this plan of treatment to most forms of zymotic disease affecting the intestines. In offensive diarrhea in adults and children, I have controlled discharges from infants almost choleraic in twelve hours by enemata 1–16000 in one-pint solutions. In cases of great irritability I have not hesitated to use chloroform carefully, to assist in controlling the child. There seems to be little need of exit tubes—the fluid is usually expelled with great force; if not, introduce them. I am treating cases of scarlet fever and diphtheria, after the acute stage has passed, by enemata, if diarrhea is present, in the belief that the colon harbors the microbe and ptomaines, and is largely chargeable with the lapses in fever and entero-colitis.

I would suggest intestinal flushing in ague, intermittents, and intestinal tuberculosis upon this theory, the only one in accord with scientific investigation and experiment. There can be no middle ground between the microbe and mystery. The invaluable labors and discoveries of bacteriologists demand of the profession from their vantage ground of opportunity corresponding zeal in the germicidal campaign. All the aids of chemistry, microscopy, hygiene and specifics are at our disposal. By modified atmospheres we can reach the ultimate air-cell, and anticipating, by early, intelligent diagnosis, organic lesions may perhaps contend successfully against the dreaded bacilli tuberculosa. Dr. Daland, of Philadelphia, who treated eleven cases of cholera at the Swinburne Island Hospital in September, 1892, in a paper read before the Philadelphia College of Surgeons, November 2, 1892, records eighty per cent. of recoveries by a treatment in which enemata containing one per cent. tannin were used with apparently specific results. A distinguished physician of Paris reports 100 cases of dysentery promptly cured by bichloride enemata. At the Military Hospital at Oran, fifty-three cases were treated with solution 1–5000 with prompt success (1891); Lemoine, of Paris (1890), fifty-four cases, 1–5000 bichloride, increasing later to 1–3000. I have not the original reports for reference, but presume they were rectal flushings where only six to eight ounces of fluid was used. I would think it hazardous to introduce two, three, or four-quart enemata 1–5000 or 1–3000. It is far better, in the present state of knowledge, to go slow. A single case of peritonitis from a rup tured ulcer, subjected to enemata by pressure or absorption of bichloride, might condemn in the minds of many an almost specific treatment, properly employed. I may err in claiming priority in the use of bichloride enemata for dysentery and typhoid, but I can find no record prior to 1883 of a case thus treated. Of course, enemata for various purposes, and charged with various substances, have been used from time immemorial, but the use of bichloride for this purpose, in the manner now described and as a germicide, I think, may not unjustly be referred to the cases cited in this paper. Further experience may prove that boric acid, tannin, carbolic acid and other antiseptics are as efficient and safer; if so, they should be preferred.

I have attached importance to special action of the bichloride solution upon the mucous membranes, observing that it was more promptly rejected than water, and conclude that even in weak solutions it has temporarily an irritating cathartic effect thus favoring its prompt expulsion. This, too, may cause the removal of tenacious mucus, and bacilli which might otherwise invade the deeper structures. I have not favored frequent large enemata when the patient was doing well, fearing that it would unfavorably affect nutrition, nor do I ignore the fact that bacteriologists concede the harmlessness of many forms of bacteria, but the morbid processes induced by others cannot be disputed. It is to the latter, when evidently present, that the most directly antiseptic treatment should be applied. It may be too much to assert that the means suggested in this paper are at once safe and sure, but if its effect be to stimulate more thorough investigation and experiment ending in a "more excellent way," I shall not feel that they have been used in vain.

